# Reduced UCP-1 Content in *In Vitro* Differentiated Beige/Brite Adipocytes Derived from Preadipocytes of Human Subcutaneous White Adipose Tissues in Obesity

**DOI:** 10.1371/journal.pone.0091997

**Published:** 2014-03-18

**Authors:** Andrew L. Carey, Camilla Vorlander, Medini Reddy-Luthmoodoo, Alaina K. Natoli, Melissa F. Formosa, David A. Bertovic, Mitchell J. Anderson, Stephen J. Duffy, Bronwyn A. Kingwell

**Affiliations:** 1 Metabolic and Vascular Physiology Laboratory, Baker IDI Heart and Diabetes Institute, Melbourne, Victoria, Australia; 2 Department of Cardiology, The Alfred Hospital, Melbourne, Victoria, Australia; Universidad Pablo de Olavide, Centro Andaluz de Biología del Desarrollo-CSIC, Spain

## Abstract

**Introduction:**

Brown adipose tissue (BAT) is a potential therapeutic target to reverse obesity. The purpose of this study was to determine whether primary precursor cells isolated from human adult subcutaneous white adipose tissue (WAT) can be induced to differentiate *in-vitro* into adipocytes that express key markers of brown or beige adipose, and whether the expression level of such markers differs between lean and obese young adult males.

**Methods:**

Adipogenic precursor cells were isolated from lean and obese individuals from subcutaneous abdominal WAT biopsies. Cells were grown to confluence, differentiated for 2.5 weeks then harvested for measurement of gene expression and UCP1 protein.

**Results:**

There was no difference between groups with respect to differentiation into adipocytes, as indicated by oil red-O staining, rates of lipolysis, and expression of adipogenic genes (*FABP4*, *PPARG*). WAT genes (*HOXC9*, *RB1*) were expressed equally in the two groups. Post differentiation, the beige adipose specific genes *CITED1* and *CD137* were significantly increased in both groups, but classic BAT markers *ZIC1* and *LHX8* decreased significantly. Cell lines from both groups also equally increased post-differentiation expression of the thermogenic-responsive gene *PPARGC1A* (PGC-1α). *UCP1* gene expression was undetectable prior to differentiation, however after differentiation both gene expression and protein content were increased in both groups and were significantly greater in cultures from lean compared with obese individuals (p<0.05).

**Conclusion:**

Human subcutaneous WAT cells can be induced to attain BAT characteristics, but this capacity is reduced in WAT cells from obese individuals.

## Introduction

Increasing brown adipose tissue (BAT) volume and activity is a potential therapeutic strategy for weight loss in obesity, which was first proposed nearly 40 years ago [1], [2]. The basis of this approach relates to the high energy consumption of activated BAT which is directed to heat production [3]. While no BAT-targeted therapies have emerged, the field has been reinvigorated in recent years by conclusive evidence of BAT presence and cold-responsive activity in adult humans [4]–[8], its responsiveness to insulin [9] and a sympathomimetic [10], impairment of function in obesity [6], [10], [11] and most recently the first conclusive evidence of adaptive BAT thermogenesis in humans [12]–[14].

Functional BAT depots in adult humans are found in the cervical, supraclavicular and various other isolated regions in the thorax and abdomen. Based on detailed study of cervical neck fat, humans probably contain a mix of white (WAT), brown and ‘intermediate’ (beige or brite adipose, hereafter referred to as beige) adipose tissue in these regions [15]. Further studies of these fat depots have revealed distinct genetic markers that may aid distinction of the different sub-classes of adipocytes in humans [16]–[19]. In contrast other fat depots in healthy humans, such as the subcutaneous abdominal region, that never exhibit cold-induced activity when imaged with ^18^F-fluorodeoxyglucose (FDG) Positron Emission Tomography (PET), are considered strictly WAT [5]–[7], [20]. Rodent WAT depots, however, have variable capacities to form beige fat, expressing high levels of BAT gene and protein markers after prolonged cold or adrenergic pharmacological stimulation [21]–[23]. Such beige depots possibly contribute to BAT thermogenesis. Thus determining whether, and the degree to which, the considerable volume of human subcutaneous WAT can form beige fat, whether by transdifferentiation or *de novo* production from precursor cells, is of importance to future therapeutic strategies for obesity.

Expression of BAT genes, particularly *UCP1*, has been reported in adult human subcutaneous abdominal WAT [24], [25]. *UCP1* can be induced in primary cultures of preadipocytes from human adults sourced from mixed WAT depots [26]–[29], and from beige/brown adipose depots [16], [17], [30], primarily through long-term treatment with the pharmacological PPARγ agonist rosiglitazone supplemented to the differentiation media. It is, however, unknown whether this tissue contains cells which can, when grown in primary culture utilizing this methodology, express beige fat-specific genes or whether this capacity differs between lean and obese humans. Using human subcutaneous WAT precursor cells from lean and obese humans grown in brown adipose differentiation media, we aimed to determine:

- their capacity to form brown/beige adipocytes by measuring expression of representative genes and UCP-1 protein.
- whether expression of brown/beige adipocyte genes and UCP-1 protein differed in cells from lean and obese individuals.

## Materials and Methods

### Participants

Nine lean (29±4 yrs, 24±1 kg/m^2^) and 8 obese (28±2 yrs, 37±2 kg/m^2^) young, healthy, sedentary and unmedicated males took part in this study, which was approved by the Alfred Hospital Ethics Committee. All patients provided written, informed consent.

### Primary human adipocyte culture

Abdominal subcutaneous adipose tissue was taken via needle biopsy (12 gauge with suction, ∼5 cm lateral to the navel). After extraction, the tissue was washed thoroughly in sterile saline and any non-adipose tissue dissected free and discarded. Preadipocytes were isolated from this tissue as described previously [31], [32], with modifications. Tissue (∼0.4 g) was placed immediately in digestion buffer (serum free alpha-MEM, 5% BSA and 3.3 mg/ml type I collagenase) on ice, then transferred within 5 min to an incubator to digested for 60 min at 37°C. The suspension was then filtered through sterile 250 μm mesh and the mature adipocytes then allowed to float to the surface of the suspension. The mature adipocytes were discarded and the fibroblastic precursor cells (the entire stromal vascular fraction) isolated and washed via centrifugation at 300×g for 10 min, and expanded in culture in Dulbecco's modified Eagle's medium (DMEM, Invitrogen; Life Technologies, Carlsbad, CA, USA) containing 1% penicillin/streptomycin (Invitrogen) and 15% fetal calf serum (FCS, Invitrogen). Similar to methods described previously for primary human skeletal muscle [33], cell lines were pooled within study groups, such that each combined cell line contained 4–5 individuals per culture. These combined cell lines were grown in culture dishes (growth area 55 cm^2^) to 80% confluence, then one dish of cells transferred to 20 separate culture wells (growth area 3.5 cm^2^). For a single experiment, one combined cell line from each of the lean and obese groups were grown in parallel, with 3–4 replicates for each condition, which represented n = 1. Each experiment was then repeated 4–5 times, as indicated in the Results section.

To differentiate cells, upon reaching confluence, cultures were left for a further 48 hours, then induced in DMEM containing 2% FCS, 1% penicillin/streptomycin, 0.5 mM isobutylmethylxanthine (Sigma-Aldrich, St Louis, MO, USA), 25 μM dexamethasone (Sigma-Aldrich), 1 μM human insulin (Sigma-Aldrich), 250 nM triiodo-L-thyronine (Sigma-Aldrich), 1 μM rosiglitazone (Cayman Chemical, Ann Arbor, MI, USA) and 250 nM indomethacin (Sigma-Aldrich) as is typical for differentiation of preadipocytes in culture to brown/beige adipocytes [17],[19],[26],[31],[34],[35]. After 7 days this media was changed to DMEM containing 2% FCS, 1% penicillin/streptomycin, 1 μM human insulin and 1 μM rosiglitazone for a further 10 days. Cells were then left for 24 hours in 2% DMEM containing no further additives prior to treatment and/or harvesting. Long-term treatment with a thioazolidinedione (rosiglitazone) as a PPARγ agonist is considered essential to induce brown-like adipocyte differentiation of human adult derived preadipocytes. The current protocol applied rosiglitazone treatment for a total of 17 days, although previous protocols have varied with regard to the use, timing and duration of rosiglitazone treatment [16], [17], [26]–[29], [36], [37]. The cell permeable 3′-5′-cyclic adenosine monophosphate analogue N^6^,2′-O-Dibutyryladenosine 3′,5′-cyclic monophosphate (db-cAMP, 0.5 mM) was used to mimic adrenergic signaling in experiments where indicated, as reported previously [38].

### Microscopy and imaging

After differentiation cells were fixed in 10% buffered formalin (Sigma-Aldrich). Cells were then stained with oil red-O and images recorded under light microscopy to examine the degree of lipid loading as a surrogate marker of adipogenesis. To quantify oil red-O staining, after imaging, cells were washed in water and air dried. Lipid-bound oil red-O was then dissolved in isopropanol at 4°C for 30 min, then the absorbance of the oil red-O in isopropanol determined at 495 nm.

### Lipolysis assay

Fresh media and treatments (PBS vehicle or 0.5 mM db-cAMP) were added to cells, which were then left for 6 hours prior to collection of the media for determination of glycerol concentration. Media glycerol concentration was quantified using a commercially available reagent (Sigma-Aldrich), measured according to manufacturer's instructions.

### Gene expression analyses

Cells were lyzed in TRIzol (Invitrogen) and total RNA extracted according to the manufacturer's directions, and as described previously [39]. The quality and quantity of the RNA were determined on a NanoDrop 1000 spectrophotometer (Nanodrop Technologies, Wilmington, DE, USA). The RNA samples were diluted as appropriate to equalize concentrations, and stored at -80°C for subsequent reverse transcription.

First-strand complementary DNA (cDNA) synthesis was performed using commercially available TaqMan Reverse Transcription kit reagents (Applied Biosystems, Melbourne, Australia) at a final concentration of 400 ng/μl. All RNA samples were reverse transcribed to cDNA in a single run from the same reverse transcription master mix. Negative control samples (no RNA or no reverse transcriptase) were run simultaneously with test samples to control for and verify that amplification did not proceed from genomic DNA and other potential contaminants.

Ten ng of cDNA was aliquoted into 96-well PCR plates and subjected to PCR analysis using a BioRad iCycler RT-qPCR system (BioRad, Gladesville, Australia). PCR with reactions contained 4 μl of cDNA template (2.5 ng/μl), 10 μl of 2 X BioRad iQ™ Supermix (BioRad), 1 μl of 20 X Taqman FAM-labelled assay-on-demand gene expression reagents, 0.5 μl of 20×18 S ribosomal RNA (rRNA) control reagents (Applied Biosystems), and DEPC H2O added to a final volume of 20 μl. Multiplex PCR conditions involved 50 cycles of 95°C for 15 sec and 60°C for 60 sec, using VIC-labelled 18S rRNA as a housekeeping gene to normalize threshold cycle (CT) values. The relative amounts of mRNAs were calculated using the comparative CT method as previously described [39]. Pilot studies indicated no induction of any adipogenic genes when cells were grown for 3 weeks in standard growth, rather than adipogenic, media. Therefore experiments and analyses were conducted by comparing post-differentiation CT with pre-differentiation values (with pre-differentiation normalized to 1). The exception to this was UCP1, for which no expression could be detected prior to differentiation, therefore comparison between groups was made using raw delta-CT values. All samples were run in duplicate simultaneously with RNA- and reverse transcriptase-negative controls.

### Western blot analyses

After differentiation cells were washed with ice-cold PBS, lysed in protein lysis buffer and western blotting performed as previously described [40]. UCP-1 protein was measured using a polyclonal antibody (Abcam, Cambridge, UK). Total beta-actin protein was quantified as an endogenous control protein (Cell Signaling, Danvers, MA, USA). Immuno-reactive bands were detected using an anti-rabbit HRP-conjugated secondary antibody (BioRad, Gladesville, NSW, Australia) followed by enhanced chemiluminescence imaging on a BioRad Geldoc XRS+ system (BioRad) and quantified on Quantity One software (BioRad). As for gene expression described above, experiments and analyses were conducted as fold-change in protein content post-differentiation with pre-differentiation values normalized to 1.

### Statistical analyses

All data are presented as mean ± SEM. Gene expression data were analysed using non-parametric Mann-Whitney U-tests, while other data were analysed via two-tailed Student's T-tests. Analyses were conducted using SPSS 15.0 or Microsoft Excel 2007 software. Significance was accepted when P<0.05.

## Results

**Figure 1:**
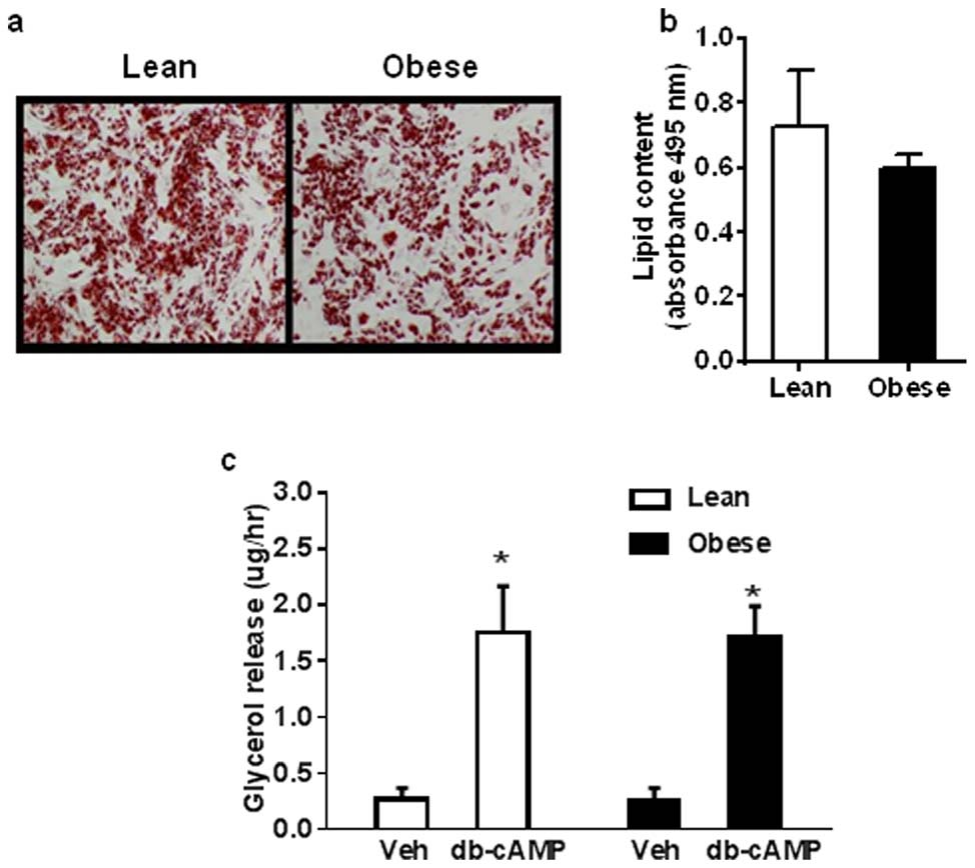
Adipogenic differentiation of cultured human primary subcutaneous white adipocye precursor cells. (a) After differentiation cells were fixed and stained with oil red-O; images shown are 10x magnification. (b) Lipid content was measured by quantification of oil red-O staining (absorbance at 495 nm) (n = 4). After differentiation cells were treated with vehicle (Veh, PBS) or 0.5 mM N^6^,2′-O-Dibutyryladenosine 3′,5′-cyclic monophosphate (db-cAMP) for 6 hours, and glycerol secretion into media was measured to determine rates of lipolysis. Glycerol secretion increased significantly with db-cAMP compared to vehicle treatment, but there was no difference between groups (*P<0.05 db-cAMP *vs* veh, n = 5).

Light microscopy (Fig 1a), and quantitation of oil red-O lipid staining (Fig 1b) indicated that cells differentiated into lipid-laden adipocytes and that there was no difference in the degree of lipid loading between cultures from lean and obese groups. Basal lipolysis (PBS vehicle) measured as the rate glycerol release from cells, was not different between groups (Fig 1c). There was a significant increase in rates of lipolysis in both groups upon stimulation for 6 hours with the cell-permeable cyclic AMP analogue, db-cAMP, (P<0.05), and this increase was similar between groups (Fig 1c).

**Figure 2:**
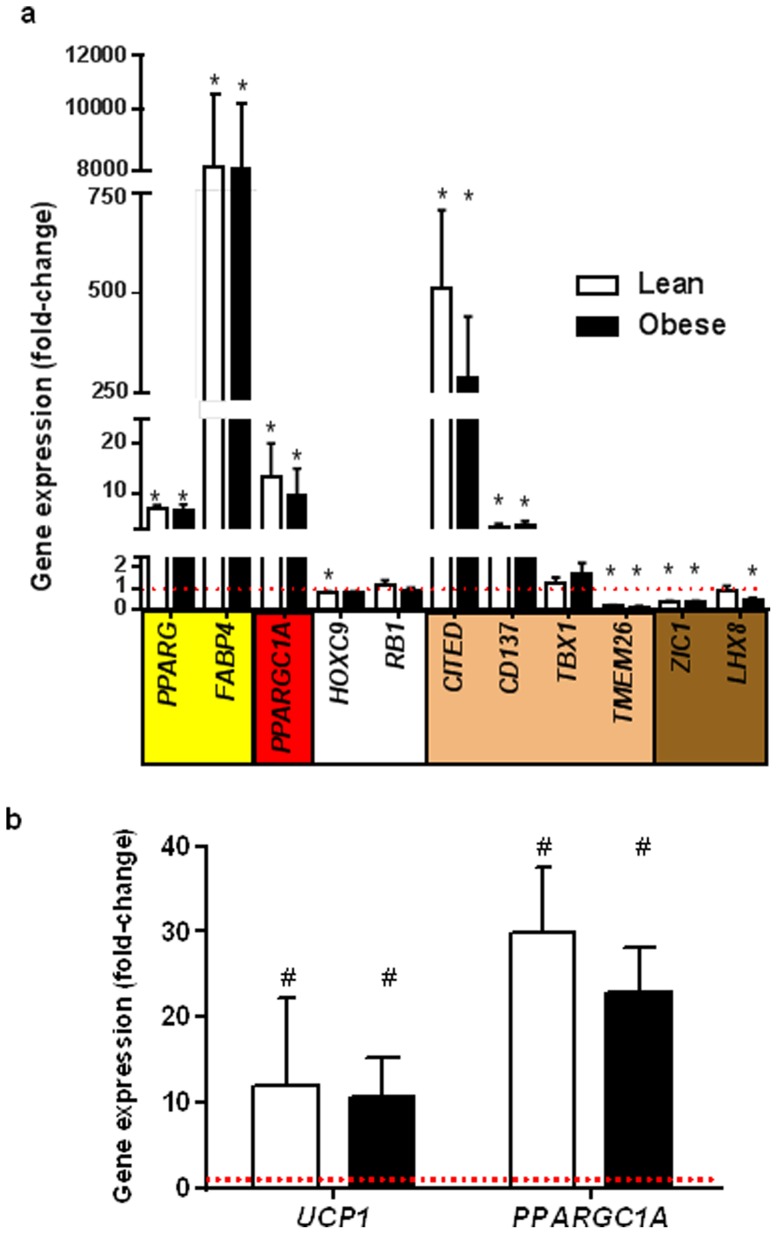
Post-differentiation expression of genes representing general adipogenesis (*PPARG*, *FABP4*; yellow highlight), general browning and thermogenesis (*PPARGC1A*; red highlight), white adipogenesis (*HOXC9*, *RB1*; white highlight), beige adipogenesis (*CITED1*, *CD137, TBX1*, *TMEM26*; beige highlight) and/or brown adipogenesis (*ZIC1*, *LHX8*; brown highlight) in cultured human primary subcutaneous white adipocye precursor cells. (a) Fold-change in expression of indicated genes post-differentiation compared with pre-differentiation (pre-differentiation expression level normalized to 1.0). (b) Fold-change in expression of indicated genes in differentiated cells after 6 h treatment with 0.5 mM db-cAMP compared with vehicle (PBS) (vehicle expression level normalized to 1.0). (n = 5, *P<0.05 pre- *vs* post-differentiation; #P<0.05 vehicle *vs* db-cAMP treatment).

Fold-changes in gene expression after differentiation compared with pre-differentiation for both groups are shown in Figure 2a. Genes that are representative of general adipogenesis (*PPARG*, lean 6.8±0.7, obese 6.6±1.0; *FABP4*, lean 7977±265, obese 8008±2336) were significantly increased after differentiation compared with pre-differentiation (P<0.05), but were not different between groups. WAT-representative genes (*HOXC9*, lean 0.79±0.07, obese 0.77±0.10; *RB1*, lean 1.14±0.25, obese 0.94±0.11) were unchanged after differentiation, although *HOXC9* expression significantly decreased in the lean group after differentiation when compared with pre-differentiation (P<0.05). Expression of *PPARGC1A*, the key factor in mitochondrial biogenesis, increased significantly and was not different between groups (lean 13.3±6.8, obese 9.5±5.5 P<0.05). Classic brown-representative genes were either unchanged or decreased after differentiation. *ZIC1* expression (lean 0.35±0.07, obese 0.38±0.05) decreased significantly in both groups, whereas *LHX8* (lean 0.87±0.26, obese 0.49±0.06) decreased only in the obese group (P<0.05). Expression of putative beige adipose markers after differentiation were disparate; *TMEM26* expression (lean 0.19±0.04, obese 0.14±0.03) [15], [16], [19], which has been recently reported to not well discriminate between brown and beige cells [18] decreased significantly in both groups, and *TBX1* (lean 1.22±0.31, obese 1.69±0.51) expression [19] was unchanged. In contrast, *CITED1* [17] (lean 511±197, obese 286±155) and *CD137* (lean 3.12±0.63, obese 3.21±1.04) [17], [19] were significantly increased equally in both groups after differentiation (P<0.05). Of note, since *UCP1* expression was not detectable before differentiation, but was expressed in all samples after differentiation, it was not possible to report fold-change data for this gene. Comparison of post-differentiation raw delta-C_T_ values, however, revealed that there was a significant decrease thus indicating an increased in *UCP1* expression (lean 21.2±0.8, obese 23.9±0.6; P<0.05).

In response to 6 hr treatment with db-cAMP, compared with PBS (vehicle), the thermogenic responsive genes *UCP1* (lean 12±10, obese 11±5) and *PPARGC1A* (lean 17±8, obese 12±5) increased significantly (P<0.05), to the same extent in both groups (Figure 2b).

**Figure 3:**
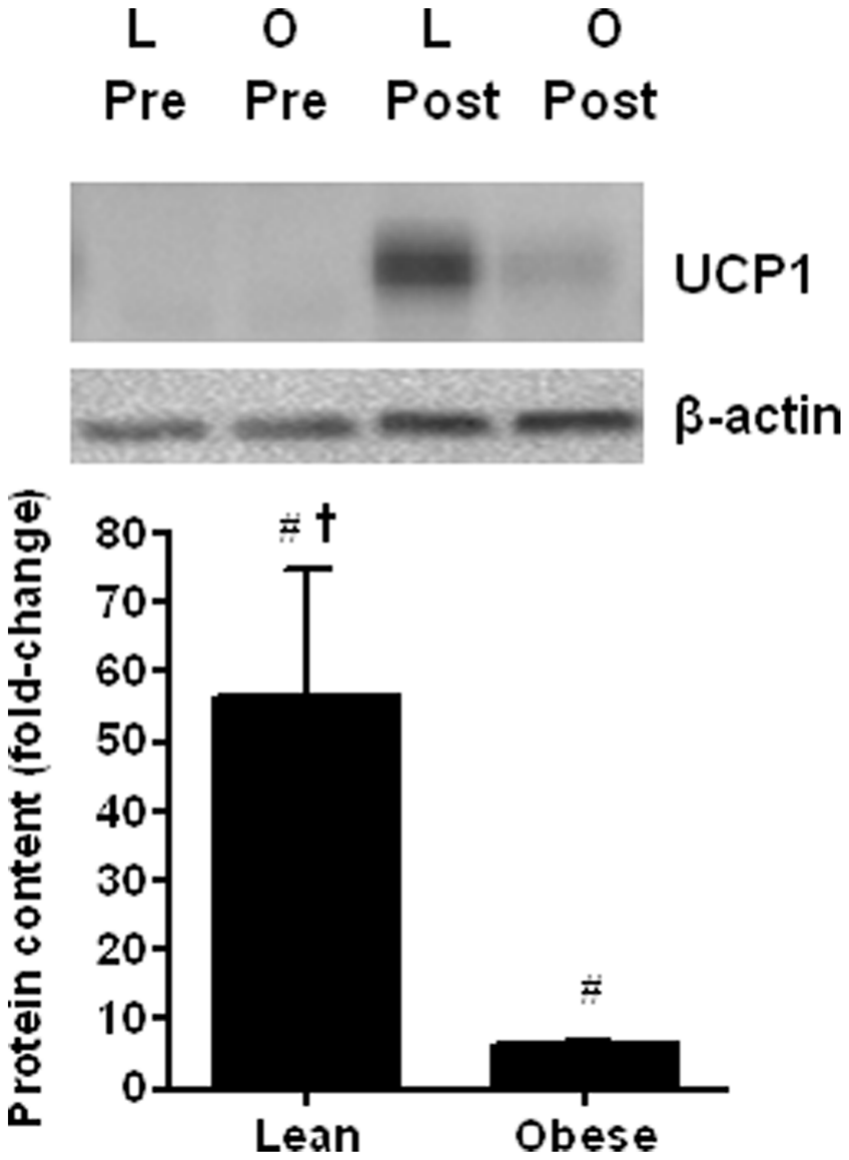
Fold-change in UCP1 protein content post-differentiation compared with pre-differentiation (n = 4, L; Lean, O; Obese; *P<0.05 vs pre-differentiation, † P<0.05 vs Obese post-differentiation, pre-differentiation normalized to 1.0).

In line with *UCP1* gene expression, UCP-1 protein content was virtually absent (although measurable via densitometry) in undifferentiated cells, and increased significantly after differentiation (lean 57±18, obese 6.5±0.6, P<0.05). Further, the fold-induction of UCP-1 protein after differentiation compared with pre-differentiation was ∼9-fold greater in cultures from lean compared with obese individuals (P<0.05, Figure 3).

## Discussion

In this study we demonstrated that primary human subcutaneous abdominal WAT contains precursor cells that can acquire brown fat characteristics based on UCP-1 gene expression and protein content. This observation is in the context of cell culture in adipogenic media containing the pharmacological PPARγ agonist rosiglitazone. Prior studies have reported that culturing of similar cells from adult humans in this manner results in browning, based on induction of *UCP1* expression [26]–[28], [41]. We now report gene expression patterns that indicate these cells are most likely a sub-population of the beige/brite adipocytes that have recently been characterized in a number of studies in humans, rather than classic brown adipocytes [15]–[17], [19]. Further, while these gene expression patterns indicate no difference between lean and obese individuals in the expression of ‘identity’ genes that characterize these cells as white, beige or brown, UCP-1 mRNA and protein content were significantly lower in cultures of cells from obese compared with lean individuals. This suggests impairment in the development of key components of the BAT thermogenic program.

Subcutaneous adipose tissue in humans is considered classic WAT. Consistent with this view, our preadipocyte cultures continually expressed *RB1*, which is involved in white adipogenesis [42], and *HOXC9*, a strong marker of human WAT [16]. Additionally, the large and significant increase in expression of genes associated with ‘general’ adipogenesis, *PPARG* and *FABP4*, are most likely driven by increases in the predominately white adipocyte population. Nevertheless, these populations clearly contain cells which can acquire BAT characteristics, although these likely represent only a small proportion of the total cell population. Small quantities of beige/brown adipose tissue have been identified in adult humans primarily in the neck, upper thoracic and paraspinal regions [4]–[8], [15], [16], [18], [19]. Given the vastly larger volume of subcutaneous WAT in humans, driving BAT-like function in this tissue is of great interest in terms of the potential to increase energy consumption. While there is evidence for transdifferentiation of mature white adipocytes into brown-like cells [43], [44] this concept is controversial. However, it is becoming well accepted that some WAT depots contain precursor cells that can form beige/brown cells upon appropriate stimulation and differentiation. Here we show that upon stimulation with media containing compounds that drive brown adipogenesis, this tissue does indeed contain beige fat precursor cells.

It is highly likely that, as in rodents, the browning process in WAT depots involves production of beige cells. Classic brown and white adipose tissues are born out of cells from distinct developmental lineages. Recent evidence supports the concept that beige adipocytes are a distinct cell type separate from classic brown and white cells, presumably from the same lineage as white adipocytes, given their location. Moreover, browning in WAT depots occurs predominately from *de novo* differentiation of beige preadipocytes, for which repeated transitions between beige- and white-ness can occur in individual cells depending on the prevailing environmental conditions [19], [45], [46]. Recent studies have revealed genes that are discriminatory for brown *vs* beige adipose in humans, with *CITED1* and *CD137* described as strong markers for beige adipocytes [17]. Further, no change or a reduction in expression of classic brown identity genes *ZIC1* and *LHX8* after differentiation, and a robust increase in expression of *CD137*, and particularly *CITED1*, is in keeping with the current literature characterizing beige adipose tissue [15]–[19]. With regard to *TMEM26* and *TBX1*, their expression clearly distinguishes beige *vs* brown adipose in mice [19], however it is not clear whether this is the case in humans [15]–[18]. Based on this, it is of relevance that our present data reflect a pattern whereby genes clearly defined as beige discriminatory (*CITED1* and *CD137*) are increased in expression, whereas those somewhat more discrepant (*TBX1* and *TMEM26*) are unchanged. This expression pattern therefore reinforces the notion that human subcutaneous WAT does contain beige, but not classic brown, precursor cells and that *CITED1* and *CD137* may be stronger beige *vs* brown adipose tissue discriminatory markers in humans than *TBX1* and *TMEM26*.

Based on the post-differentiation similarity between lean and obese individuals with respect to either brown or beige ‘identity’ genes (*CITED1, TBX1, CD137, TMEM26*, *ZIC1*, *LHX8*), and induction of *PPARGC1A* and *UCP1* upon mimicking acute adrenergic signaling, there may be no difference in the number of precursor cells, capacity to differentiate or respond to acute adrenergic signals in obese individuals. However given the use of the potent PPARγ agonist rosiglitazone in the differentiation media, we may have overridden and therefore masked any potential difference in adipogenic regulatory processes that may be present between the two groups. Accordingly, our finding of reduction in UCP-1 gene and protein expression may be indicative of diminished capacity for facultative and adaptive thermogenesis, where in response to adrenergic signaling brown/beige fat increases energy expenditure acutely (facultative thermogenesis) and UCP-1 content and maximal oxidative capacity after chronic stimulation (adaptive thermogenesis). These data are in line with human *in vivo* interventional studies that universally demonstrate a reduction in the ability to activate BAT in obese individuals [5], [6], [10], [11], [47]. Whether such defects might contribute casually to obesity, or rather, are a consequence, is unknown.

It is interesting to note that we observed disparity in UCP-1 mRNA and protein content. Specifically, while both increased significantly after differentiation in both groups of cells, it is apparent that the difference in protein content between groups (∼9-fold) was greater than the change in mRNA level (a difference in delta-C_T_ of ∼2 corresponds to a difference in copy number of ∼4-fold). The concept that the accumulation of mRNA and protein for UCP-1 follow different chronologies has been elegantly described by Nedergaard and Cannon [48]. In mice *in vivo* relative (per unit tissue mass) *Ucp1* transcription rises rapidly in response to thermogenic stimuli, then rapidly falls within a few days, whereas relative UCP-1 protein content begins to increase after this point [48]. Further time-course experiments would be required to establish whether this pattern is maintained throughout the development of these cultures, and to provide insight regarding the nature of the defect in the obese group. Regardless, it appears that certain regulatory processes are impaired in both UCP-1 transcription and translation in obesity.

Previous human studies have shown human WAT depots express markers of BAT [24], [25]. Primary cultures of human white fat can express *UCP1* with adenoviral-mediated over-expression of PPARGC1A/PGC-1α [49], or from a mixed primary tissue source [26]–[29] when grown in a brown adipogenic media cocktail. Here, we demonstrate that induction of browning in pure subcutaneous abdominal fat cells grown in similar media, containing rosiglitazone, is possible in the absence of exogenously delivered gene vectors. While these cells do not differentiate without addition of such media, in particular a PPARγ agonist (ie, rosiglitazone), browning of such cells in the presence of rosiglitazone but in the absence of the transcription factor PRDM16 is limited [50]. This and the present data therefore support the notion that human subcutaneous WAT does contain preadipocytes with capacity to differentiate into beige adipocytes, and that this result is not simply a by-product of a non-physiological media cocktail.

### Strengths and Limitations

This study examined cultures of cells taken from well characterized lean and obese individuals, but otherwise healthy individuals. As such, we sampled tissue via minimally invasive needle biopsy and therefore did not obtain numbers of cells that would allow sorting and further sub-culture of specific adipose cell populations. Sub-culture may have unmasked differences in adipogenic characteristics in brown/beige populations which were undetectable in our mixed culture approach where white adipocytes dominate. However, the use of pooled cultures from multiple individuals, permitted growth and differentiation from only a small number of cells and served to minimized biological variation. Furthermore, this approach demonstrated expression of brown *vs* beige gene markers after differentiation and identified differences between cells derived from lean and obese individuals.

In conclusion, we have provided evidence that subcutaneous human WAT contains beige, but not brown adipose progenitor cells that can be grown in primary culture without exogenous delivery of vectors to over-express browning factors. In addition, upon differentiation, induction of a complete thermogenic program is impaired in cells from obese individuals. This finding suggests that in addition to the current focus on increasing the volume and function of the well characterized brown and beige fat depots in the deep thoracic and neck regions to reverse obesity, progenitors in WAT depots should also be a focus. Further studies to better characterize different WAT sites in humans are warranted.
